# Effects of Inhaled Corticosteroids and Long-Acting β2-Agonists on Efferocytosis and Inflammatory Cell Survival: An In Vitro Study Relevant to COPD and Lung Cancer

**DOI:** 10.3390/ijms27104627

**Published:** 2026-05-21

**Authors:** Bassam Redwan, Christian Biancosino, Stefan Fischer, Sabina Janciauskiene, Heiko Golpon

**Affiliations:** 1 Department of Thoracic Surgery, Knappschaft Kliniken Gelsenkirchen Buer, Schernerweg 4, 45894 Gelsenkirchen, Germany; bassam.redwan@gmail.com; 2 Helios University Hospital Wuppertal, University of Witten/Herdecke, 42283 Wuppertal, Germany; christian.biancosino@helios-gesundheit.de; 3 Department of Thoracic Surgery and Lung Assist, Klinikum Ibbenbüren, 49477 Ibbenbüren, Germany; s.fischer@mathias-stiftung.de; 4 Department of Respiratory Medicine, Hannover Medical School (MHH), Carl-Neuberg-Straße 1, 30625 Hannover, Germany; janciauskiene.sabina@mh-hannover.de; 5 Biomedical Research in End-Stage and Obstructive Lung Disease (BREATH), German Center for Lung Research (DZL), 30625 Hannover, Germany

**Keywords:** efferocytosis, phagocytosis, inhaled corticosteroids, fluticasone propionate, salmeterol xinafoate, long-acting β2 agonists, COPD, lung cancer, tumor microenvironment, macrophages, neutrophils, apoptosis

## Abstract

Efferocytosis—the tightly regulated clearance of apoptotic cells by phagocytes—maintains tissue homeostasis and is impaired in chronic obstructive pulmonary disease (COPD), where it contributes to persistent inflammation and increases the risk of comorbidities, including lung cancer. Inhaled corticosteroids (ICS) and long-acting β2 agonists (LABAs) are cornerstones of COPD therapy, but their effects on efferocytosis and on the COPD–lung cancer interface are incompletely understood. The primary objective of this study was to determine whether the ICS fluticasone propionate and the LABA salmeterol xinafoate, alone or in combination at clinically informed concentrations (10^−8^–10^−6^ M; 10^−4^ M reserved for cytotoxicity screening), modulate efferocytic capacity and inflammatory cell survival across diverse phagocyte models. We performed standardized in vitro efferocytosis assays using murine peritoneal and alveolar macrophages, the murine macrophage line J774A.1, PMA-differentiated human THP-1 macrophages, human blood-derived neutrophils, and the human alveolar adenocarcinoma cell line A549. Apoptosis was induced in Jurkat T cells by UV irradiation (100 mJ/cm^2^) and in murine thymocytes by dexamethasone (1 µM, 4 h); apoptotic and necrotic populations were characterized by annexin-V/propidium iodide and Sytox Green/Hoechst H-33342 staining. Peritoneal macrophages showed the highest efferocytic activity (~75%), followed by J774A.1 (~75% at 24 h), THP-1 (~30% at 2 h; ~60% at 24 h), alveolar macrophages (~40%), and A549 cells (<20%). Neither fluticasone nor salmeterol, individually or in combination, significantly altered efferocytic capacity in any phagocyte tested (all ANOVA *p* > 0.26). Fluticasone (10^−8^ and 10^−6^ M) significantly improved 24 h neutrophil survival and reduced early apoptosis (*p* < 0.05) but did not translate this survival benefit into enhanced efferocytosis. Salmeterol was cytotoxic at 10^−4^ M and inactive at 10^−8^–10^−6^ M. These findings indicate that the established anti-inflammatory benefits of ICS/LABA in COPD do not extend to augmentation of efferocytosis in this acute, serum-free in vitro setting and that pharmacological restoration of efferocytosis in COPD—a defect implicated in the pathogenesis and progression of comorbid lung cancer—will likely require strategies targeting the efferocytic machinery itself (e.g., MerTK, Rac-1, MFG-E8) rather than relying on current inhaled therapy.

## 1. Introduction

Chronic obstructive pulmonary disease (COPD) is a progressive inflammatory lung disease characterized by persistent airway inflammation and impaired resolution of immune responses, including defective efferocytosis [1,2]. This failure of inflammation resolution drives ongoing tissue injury and increases the risk of comorbidities, most notably lung cancer [3,4].

The tumor microenvironment (TME) is sustained by the same chronic, unresolved inflammation that characterizes COPD [5] and has long been described as “a wound that does not heal” [6]. Persistent inflammatory signaling recruits cytokines, chemokines, and effector cells [7,8] that engage shared pathways to support tumor growth, invasion, and metastasis [9,10]. Neutrophils, in particular, play context-dependent roles in tumor biology: tumor-associated neutrophils can promote angiogenesis, suppress anti-tumor immunity, and facilitate metastatic dissemination, while in other settings they exert cytotoxic anti-tumor activity [5,11].

### 1.1. Defective Efferocytosis in COPD

COPD is highly prevalent in patients with non-small cell lung cancer (NSCLC) and is associated with worse clinical outcomes [4,12,13,14,15]. A central mechanistic link between COPD and lung cancer is defective efferocytosis in the lung. Alveolar macrophages from patients with COPD show markedly reduced uptake of apoptotic airway epithelial cells compared with healthy controls (~12% vs. ~26%), whereas general phagocytosis of inert particles remains intact—indicating a specific defect at the level of efferocytic receptor signaling rather than a global phagocytic impairment [2].

Recent evidence indicates that impaired efferocytosis is a consistent feature of COPD that persists even after smoking cessation and may represent a core, durable disease mechanism [16]. The defect spans multiple steps of the efferocytosis pathway, including reduced receptor expression (e.g., MerTK), decreased bridging molecules, and impaired intracellular signaling [16]. Failure to clear apoptotic cells results in secondary necrosis, release of damage-associated molecular patterns (DAMPs), and sustained inflammation, perpetuating airway destruction [3].

### 1.2. Apoptosis and Efferocytosis in the Tumor Microenvironment

Apoptosis is essential for tissue homeostasis and constitutes a major barrier to tumor development [17]. Apoptotic cells release “find-me” signals and expose “eat-me” signals such as phosphatidylserine, which are recognized by macrophage receptors (e.g., MerTK, Tyro3, Axl) directly or via bridging molecules including MFG-E8, Gas6, and Protein S [18,19]. Healthy cells display “don’t-eat-me” signals such as CD47 [20], and the dynamic balance among these signals determines whether cells are cleared rapidly or persist to drive inflammation. Resolution of inflammation by efferocytosis is also under transcriptional and epigenetic control: recent work demonstrates that the Notch effector RBPJ acts as a master epigenetic regulator that couples efferocytic engagement to a pro-resolving macrophage transcriptional program [21].

In the TME, therapy-induced apoptosis can paradoxically promote tumor progression when clearance is inefficient [22]. Tumor-associated macrophages (TAMs) that engulf apoptotic tumor cells acquire an immunosuppressive, pro-tumor phenotype and support metastasis [23]. When efferocytic capacity is overwhelmed, apoptotic cells progress to secondary necrosis, amplifying inflammation and tumor growth [24].

### 1.3. Efferocytosis in the TME—A Double-Edged Sword

Efferocytosis of apoptotic tumor cells by TAMs promotes immunosuppression [19,25]. MerTK signaling induces anti-inflammatory cytokines (IL-10, TGF-β), suppresses anti-tumor immunity, and supports tumor progression and metastasis. High MerTK activity correlates with poor prognosis, whereas its pharmacological inhibition restores anti-tumor responses, particularly when combined with immune checkpoint blockade [26]. Genetic or pharmacological targeting of MerTK reduces efferocytosis and suppresses tumor growth [27], establishing it as a promising therapeutic target.

### 1.4. Glucocorticoid-Augmented Efferocytosis

Glucocorticoid-augmented efferocytosis (GCAE) refers to the ability of glucocorticoids to enhance macrophage clearance of apoptotic cells. Glucocorticoids upregulate MerTK and engage downstream pathways (LXR, PPARδ) that promote efficient apoptotic-cell removal [28,29]. This effect, however, has a clinically important trade-off: enhanced efferocytosis can blunt bactericidal function and increase susceptibility to respiratory infection, providing a mechanistic explanation for the elevated pneumonia risk observed in patients with COPD receiving inhaled corticosteroids [30].

### 1.5. Mechanistic Rationale for Studying ICS and LABA on Phagocyte Function

Inhaled corticosteroids modulate phagocyte biology through both genomic and non-genomic mechanisms. Genomically, ICSs bind cytoplasmic glucocorticoid receptors, translocate to the nucleus, and modulate transcription of efferocytosis-related genes (e.g., *MERTK*, *PPARG*, *LXR*) and survival genes (e.g., Mcl-1, Bcl-xL) [28,29,31]. Non-genomically, ICSs rapidly inhibit caspase activation and stabilize anti-apoptotic mitochondrial signaling in neutrophils [31]. β2 agonists act through cAMP/PKA signaling and can potentiate corticosteroid-induced anti-apoptotic effects in neutrophils [32]. Despite extensive characterization of these pathways in single cell systems, the *net* effect of clinically relevant ICS/LABA combinations on efferocytic clearance—particularly across the heterogeneous phagocyte populations encountered in the COPD lung and the TME—has not been systematically examined.

### 1.6. Clinical Gap and Study Objectives

LABAs and ICSs are cornerstones of COPD pharmacotherapy, and their combination confers additional anti-inflammatory benefit beyond either agent alone [33,34]. However, β2 agonists may potentiate corticosteroid-induced neutrophil survival, raising concern that prolonged neutrophil persistence in the airway—without a corresponding improvement in efferocytosis—could accelerate secondary necrosis and inflammatory tissue injury [32]. Clinical data further indicate that salmeterol/fluticasone differentially modulate airway inflammatory cell populations, reducing T-cell and macrophage numbers while exerting variable effects on neutrophils [35].

Whether these clinically established anti-inflammatory effects translate into improved apoptotic-cell clearance—the key homeostatic process whose failure links COPD to lung cancer pathogenesis—remains an unresolved clinical gap. The primary objective of this study was therefore to determine, under standardized in vitro conditions, whether fluticasone propionate and salmeterol xinafoate, alone or in combination, modify (i) efferocytic capacity across diverse professional and non-professional phagocyte models and (ii) the survival of human blood neutrophils. The novelty of our approach lies in the breadth of phagocyte models examined within a single standardized assay platform (murine peritoneal macrophages, murine alveolar macrophages, J774A.1, PMA-differentiated THP-1, human blood neutrophils, and A549 lung epithelial cells) and in the use of two independent apoptotic target populations (UV-induced apoptotic Jurkat T cells and dexamethasone-induced apoptotic murine thymocytes), enabling direct cross-model comparison of efferocytic responses to a clinically relevant ICS/LABA combination.

## 2. Results

### 2.1. Cytotoxicity Screening of Fluticasone, Salmeterol, and Their Combination in THP-1 Macrophages

**Figure 1:**
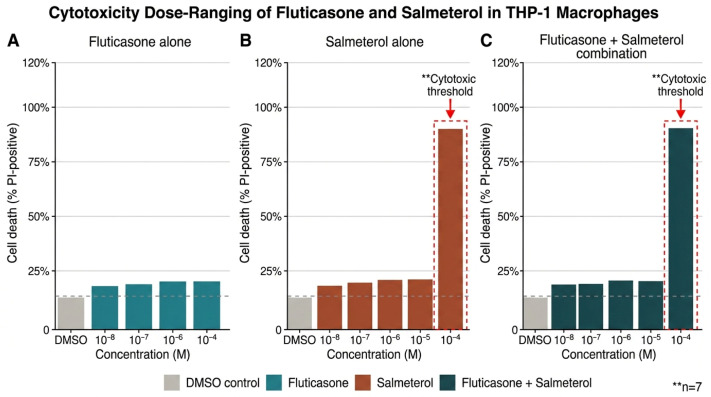
PMA-differentiated THP-1 macrophages were treated with (**A**) fluticasone, (**B**) salmeterol, or (**C**) fluticasone + salmeterol in combination at 10^−8^–10^−4^ M for 24 h (n = 2–3 per concentration). Dead cells were enumerated by fluorescence microscopy after propidium iodide staining. Salmeterol at 10^−4^ M caused marked cytotoxicity, an effect preserved in the combination but absent with fluticasone alone. Concentrations of 10^−8^–10^−5^ M were non-cytotoxic.

Cell viability assays were performed to define safe working concentrations of fluticasone and salmeterol (Figure 1). PMA-differentiated THP-1 macrophages were treated for 24 h with each drug alone or in combination over a five-log concentration range (10^−8^–10^−4^ M), and viability was assessed by propidium iodide exclusion. Salmeterol produced clear cytotoxicity at 10^−4^ M, an effect retained in the fluticasone + salmeterol combination but absent with fluticasone alone. No cytotoxicity or morphological changes were observed between 10^−8^ and 10^−5^ M. On this basis, working concentrations of 10^−8^–10^−6^ M were used in all subsequent functional assays—a range that brackets the local epithelial-lining-fluid concentrations achieved with clinically used inhaled doses of fluticasone propionate (estimated 10^−7^–10^−6^ M) and salmeterol xinafoate (10^−9^–10^−7^ M) [36,37].

### 2.2. Standardized Induction and Characterization of Apoptotic Target Cells

**Figure 2:**
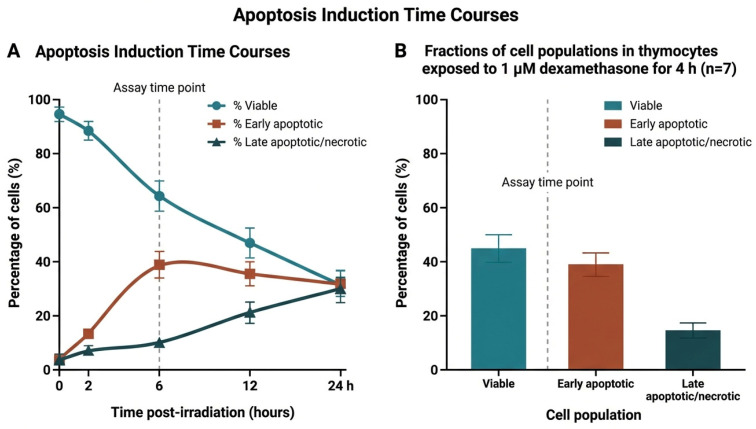
(**A**) Jurkat T cells were exposed to UV irradiation (100 mJ/cm^2^), and the proportions of viable, apoptotic, and necrotic cells were quantified by fluorescence microscopy at 2, 6, and 24 h after irradiation following annexin-V/propidium iodide staining (n = 3 independent experiments). (**B**) Murine thymocytes were treated with dexamethasone (1 µM) for 4 h, and viable, apoptotic, and necrotic cells were quantified as in (**A**) (n = 7 independent experiments).

Apoptosis was induced in Jurkat T cells by UV irradiation (100 mJ/cm^2^) and in murine thymocytes by dexamethasone (1 µM, 4 h). Time-course analysis confirmed the generation of reproducible populations of early apoptotic, late apoptotic, and necrotic cells, validating their use as standardized targets for efferocytosis assays (Figure 2A,B).

### 2.3. Effect of Fluticasone and Salmeterol on Jurkat T-Cell Viability and Inducibility of Apoptosis

**Figure 3:**
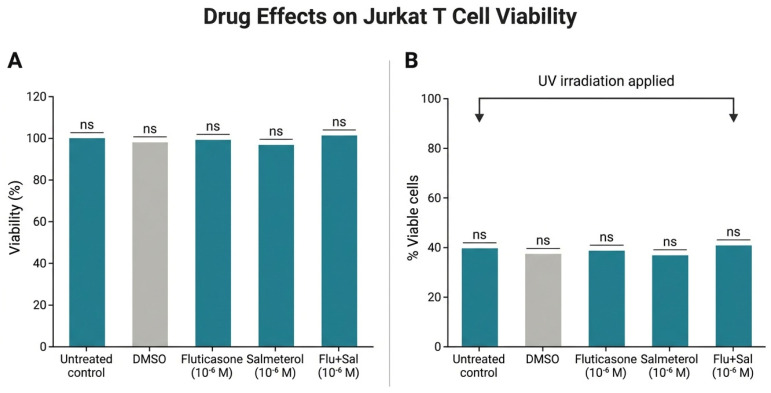
(**A**) Jurkat T cells were treated with fluticasone (Flu), fluticasone + salmeterol (Flu + Sal), or salmeterol (Sal) at 10^−6^ M for 4 h, and viability was quantified by flow cytometry after annexin-V/propidium iodide staining (n = 3). None of the treatments altered Jurkat viability. (**B**) Jurkat T cells pre-treated as in (**A**) were UV-irradiated to induce apoptosis. After 24 h, viability was quantified by flow cytometry (n = 3); none of the treatments prevented UV-induced cell death. Statistical analysis: n.s., not significant (*p* ≥ 0.05).

To exclude direct effects of test compounds on the apoptotic target population, we assessed whether fluticasone and salmeterol modified Jurkat T-cell viability (Figure 3A). At 10^−6^ M, neither fluticasone, salmeterol, nor their combination altered baseline Jurkat viability. Furthermore, none of the treatments prevented UV-induced apoptosis (Figure 3B), confirming the suitability of UV-irradiated Jurkat cells as standardized apoptotic targets independent of drug treatment.

### 2.4. Fluticasone Prolongs Neutrophil Survival in a Concentration-Dependent Manner

**Figure 4:**
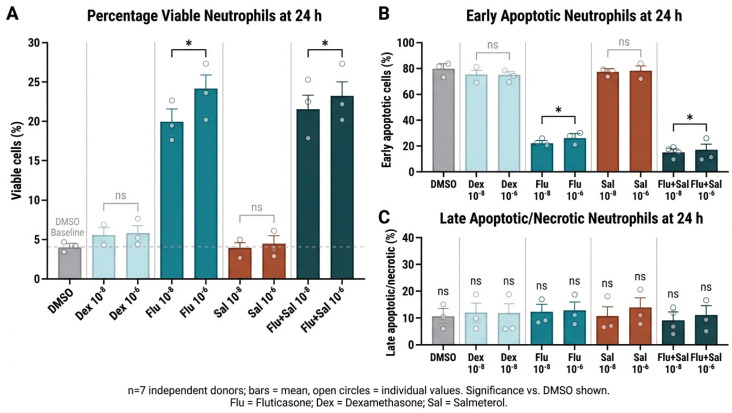
Human peripheral-blood neutrophils were treated with dexamethasone (Dex), fluticasone (Flu), fluticasone + salmeterol (Flu + Sal), or salmeterol (Sal) at 10^−8^ M or 10^−6^ M for 24 h (n = 7 independent donors). (**A**) Viable cells (annexin-V^−^/propidium iodide^−^) were quantified by flow cytometry. Fluticasone (10^−8^ M and 10^−6^ M) and the fluticasone + salmeterol combination significantly increased viability versus vehicle (ANOVA with Student–Newman–Keuls, *p* < 0.05); dexamethasone produced only a non-significant trend. (**B**) Early apoptotic cells (annexin-V^+^/propidium iodide^−^) were significantly reduced by fluticasone and by fluticasone + salmeterol. (**C**) Late apoptotic/necrotic cells (annexin-V^+^/propidium iodide^+^) did not differ significantly between treatments. Statistical analysis: * *p* < 0.05 vs. vehicle control; n.s., not significant; one-way ANOVA with Stu-dent–Newman–Keuls post-hoc test.

After 4 h in culture, more than 90% of freshly isolated human blood neutrophils remained viable, with only a small fraction undergoing apoptosis or necrosis. At this early time point, treatment with dexamethasone, fluticasone, salmeterol, or fluticasone + salmeterol (10^−8^–10^−6^ M) had no detectable effect on viability or cell death (ANOVA, *p* > 0.88). After 24 h in vehicle-treated cultures, neutrophil viability fell to less than 5%, with approximately 75% of cells appearing as early apoptotic and 20% as late apoptotic or necrotic (Figure 4). Fluticasone significantly increased viability and decreased the proportion of early apoptotic cells at both 10^−8^ M and 10^−6^ M (ANOVA with Student–Newman–Keuls, *p* < 0.05). The fluticasone + salmeterol combination produced a similar survival effect, with no additional benefit beyond fluticasone alone. Salmeterol monotherapy had no effect, and dexamethasone caused only a small, non-significant increase in viability. No treatment altered the proportion of late apoptotic or necrotic cells (Figure 4C).

### 2.5. Efferocytosis of Apoptotic Cells by Murine Alveolar and Peritoneal Macrophages

**Figure 5:**
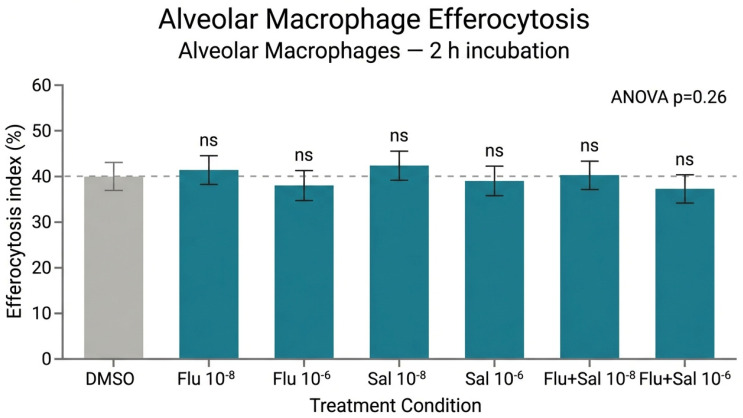
Unstimulated alveolar macrophages from female C57BL/6 mice were co-incubated for 2 h with dexamethasone-induced apoptotic murine thymocytes (apo Thy). Engulfment in response to fluticasone (Flu), fluticasone + salmeterol (Flu + Sal), or salmeterol (Sal) at 10^−8^ M or 10^−6^ M (n = 4) was quantified by fluorescence microscopy. ANOVA revealed no significant differences between treatments (*p* = 0.26). Statistical analysis: n.s., not significant (*p* ≥ 0.05, one-way ANOVA).

**Figure 6:**
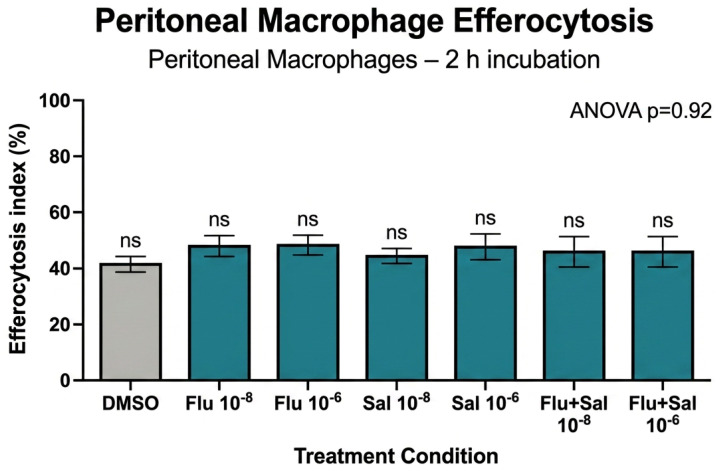
Unstimulated peritoneal macrophages from female C57BL/6 mice were co-incubated for 2 h with apoptotic thymocytes (apo Thy) as in Figure 5. Engulfment in response to fluticasone, fluticasone + salmeterol, or salmeterol at 10^−8^ M or 10^−6^ M (n = 3) was quantified by fluorescence microscopy. ANOVA revealed no significant differences between treatments (*p* = 0.92). Statistical analysis: n.s., not significant (*p* ≥ 0.05, one-way ANOVA).

After 2 h of co-incubation with apoptotic thymocytes, approximately 40% of unstimulated murine alveolar macrophages had engulfed apoptotic bodies (Figure 5). Fluticasone and salmeterol at 10^−8^ M and 10^−6^ M produced a slight, non-significant increase in uptake (ANOVA, *p* = 0.26). Peritoneal macrophages displayed substantially higher efferocytic capacity (~75%) than alveolar macrophages (~40%) over the same 2-h period (Figure 6), and neither fluticasone nor salmeterol modified peritoneal-macrophage efferocytosis (ANOVA, *p* = 0.92). Quantitative efferocytosis data across all phagocyte models are summarized in Table 1.

### 2.6. Efferocytosis of Apoptotic Cells by J774A.1 and PMA-Differentiated THP-1 Macrophages

**Figure 7:**
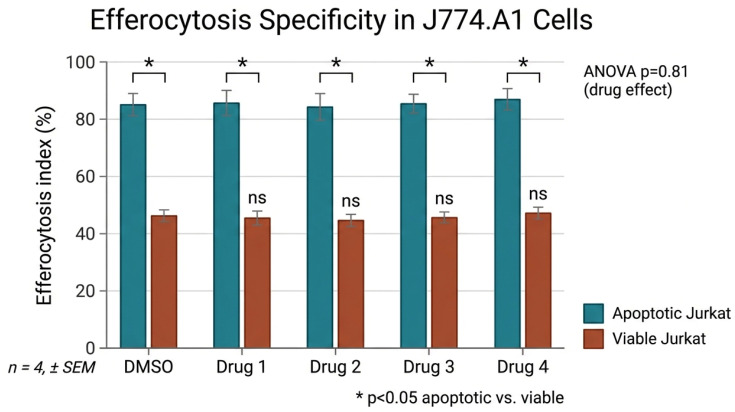
J774A.1 murine macrophages were co-incubated with UV-induced apoptotic Jurkat T cells (apo Ju) for 24 h. Engulfment in response to fluticasone (Flu), fluticasone + salmeterol (Flu + Sal), or salmeterol (Sal) at 10^−8^ M or 10^−6^ M (n = 4) was quantified by fluorescence microscopy. ANOVA revealed no significant differences between treatments. Statistical analysis: n.s., not significant (*p* ≥ 0.05, one-way ANOVA).

After 24 h of co-incubation with UV-induced apoptotic Jurkat T cells, approximately 75% of J774A.1 macrophages had engulfed apoptotic targets (Figure 7), and efferocytosis was not modified by fluticasone, salmeterol, or their combination. Preference for apoptotic over viable Jurkat targets confirmed assay specificity.

**Figure 8:**
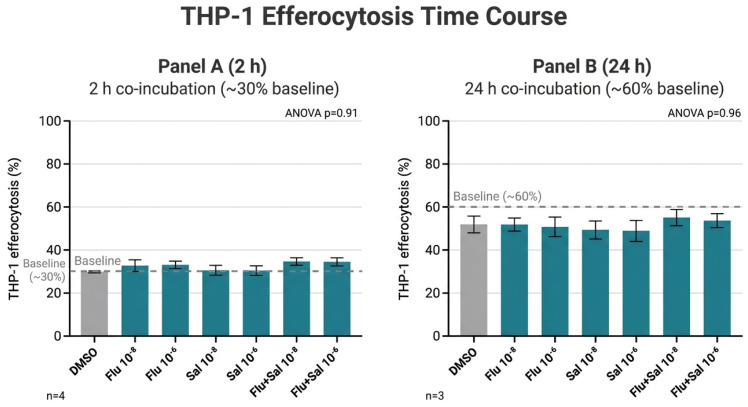
PMA-differentiated THP-1 macrophages were co-incubated with UV-induced apoptotic Jurkat T cells (apo Ju) for (**A**) 2 h (n = 4) or (**B**) 24 h (n = 3). Engulfment in response to fluticasone, fluticasone + salmeterol, or salmeterol at 10^−8^ M or 10^−6^ M was quantified by fluorescence microscopy. ANOVA revealed no significant differences between treatments at either time point.

In PMA-differentiated THP-1 macrophages, approximately 30% of cells had engulfed apoptotic Jurkat targets after 2 h, increasing to approximately 60% after 24 h (Figure 8A,B). Neither time point showed a treatment effect (ANOVA, *p* = 0.91–0.96).

### 2.7. Efferocytosis of Apoptotic Cells by A549 Lung Epithelial Cells (Non-Professional Phagocytes)

**Figure 9:**
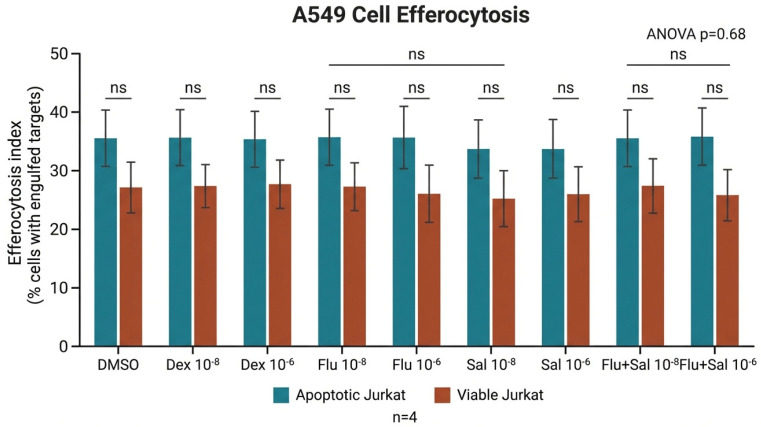
A549 lung epithelial cells were co-incubated for 24 h with viable (via) or apoptotic (apo) Jurkat T cells (Ju). Engulfment in response to dexamethasone (Dex), fluticasone (Flu), fluticasone + salmeterol (Flu + Sal), or salmeterol (Sal) at 10^−8^ M or 10^−6^ M (n = 4) was quantified by fluorescence microscopy. ANOVA revealed no significant differences between treatments (*p* = 0.68–0.88). Statistical analysis: n.s., not significant (*p* ≥ 0.05, one-way ANOVA).

**Figure 10:**
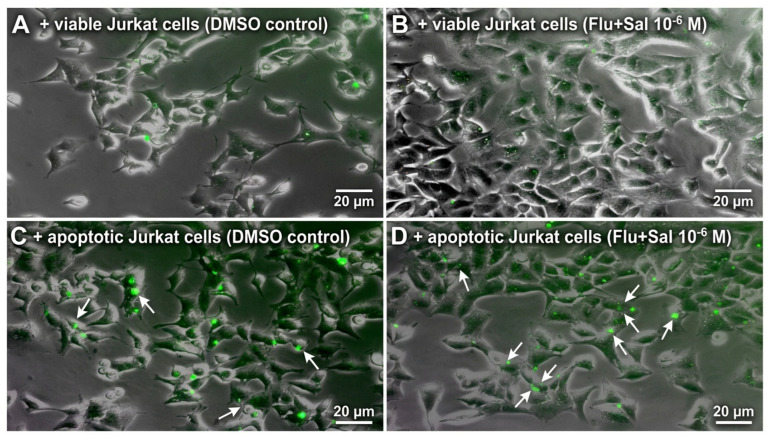
A549 cells were co-incubated for 24 h with Calcein AM-labeled viable Jurkat T cells (**A**,**B**) or apoptotic Jurkat T cells (**C**,**D**); cells were then washed three times with PBS. Green fluorescence indicates Jurkat cells attached to or engulfed by A549 cells. Fluticasone + salmeterol treatment (**B**,**D**) did not alter the number of attached or engulfed Jurkat cells compared with vehicle (**A**,**C**). Arrows indicate engulfed apoptotic Jurkat T cells (apo Ju) within A549 epithelial cells, visualised by Calcein-AM fluorescence. Scale bars and magnification as indicated.

After 24 h of co-incubation with apoptotic Jurkat T cells, fewer than 20% of A549 cells had engulfed apoptotic targets (Figure 9). Efferocytosis was not modified by fluticasone, salmeterol, their combination, or dexamethasone (ANOVA, *p* = 0.68–0.88). Minimal uptake of viable Jurkat targets confirmed apoptotic-cell specificity. Representative fluorescence-microscopy images of A549 co-incubations with viable and apoptotic Jurkat T cells are shown in Figure 10.

### 2.8. Summary of Efferocytic Activity Across Phagocyte Models

Table 1 summarizes baseline efferocytic activity and the effect of fluticasone, salmeterol, and fluticasone + salmeterol across all phagocyte models. Across professional and non-professional phagocytes, no ICS/LABA treatment significantly modified efferocytic capacity (all ANOVA *p* > 0.26).

## 3. Discussion

The clinical relevance of COPD as a comorbidity of lung cancer is now firmly established: COPD is independently associated with increased lung-cancer incidence and worse oncological outcomes after surgical resection and systemic therapy [4,38]. Shared pathogenic features include epithelial–mesenchymal transition (EMT) and chronic, unresolved inflammation [39,40]. Within this nexus, defective efferocytosis has emerged as a mechanistic axis that connects sustained airway inflammation, secondary necrosis, and an immunosuppressive lung microenvironment permissive to tumor initiation and progression [3,16,22].

### 3.1. Phagocyte Heterogeneity and Efferocytic Capacity

Our data confirm a hierarchy of efferocytic capacity: murine peritoneal macrophages (~75%) > J774A.1 (~75% at 24 h) > PMA-differentiated THP-1 (~60% at 24 h) > murine alveolar macrophages (~40%) > A549 alveolar epithelial cells (<20%). This pattern is consistent with the literature and reflects the functional adaptation of macrophages to their local microenvironment [41]. Alveolar macrophages, although the first-line cellular defense of the alveolar space, express comparatively low levels of several efferocytosis receptors and are subject to inhibitory regulation by surfactant proteins A and D via SIRPα [30]. We use the term “functional adaptation” deliberately: although our serum-free, ex vivo assay cannot demonstrate tissue-resident specialization in situ, the magnitude and reproducibility of the inter-population differences observed here are concordant with single-cell transcriptomic and proteomic profiles of peritoneal versus alveolar macrophages published elsewhere [41]. In the context of COPD and lung cancer, this comparatively limited efferocytic reserve of alveolar macrophages assumes particular pathological importance: the very phagocyte population most exposed to apoptotic cellular debris in the airway is the one with the lowest baseline clearance capacity.

### 3.2. Glucocorticoids, Efferocytosis, and the Acute In Vitro Paradox

Fluticasone did not enhance efferocytosis in any phagocyte type studied, in apparent contrast to reports that glucocorticoids increase apoptotic-cell clearance. Prior work has shown that GCAE depends critically on serum-derived opsonins (Protein S → MerTK signaling) and on prolonged exposure that engages transcriptional programs downstream of LXR and PPARδ [28,29]. The discrepancy between our results and previous reports most likely reflects three deliberate features of our experimental design: (i) assays were performed in serum-free X-Vivo medium to permit fluorescence-based readouts, thereby limiting opsonin-dependent mechanisms; (ii) drug exposure was acute (2–24 h), which may be insufficient to engage the slower transcriptional arms of glucocorticoid action; and (iii) phagocytes were derived from healthy donors and animals, which already exhibit high baseline efferocytic capacity and offer a smaller dynamic range for further augmentation.

Importantly, fluticasone is a class-representative ICS rather than a unique entity: its anti-inflammatory pharmacology—including upregulation of MerTK and modulation of LXR/PPARδ—is shared with other inhaled glucocorticoids such as budesonide and beclomethasone [28,29,31]. Our negative findings for fluticasone in this acute, serum-free model should therefore be interpreted as ICS class effects on acute in vitro efferocytosis rather than as agent-specific properties of fluticasone propionate. Lung deposition characteristics differ between ICS molecules (e.g., budesonide is more hydrophilic than fluticasone), and these clinical-pharmacology differences may shape the in vivo relevance of GCAE in ways that our in vitro system cannot resolve.

Taken together, these considerations indicate that glucocorticoid effects on efferocytosis are highly context-dependent and may emerge more clearly under in vivo or chronic-treatment conditions, consistent with the elevated pneumonia risk observed in patients with COPD receiving ICS [30]. We did not assess efferocytic-receptor expression (MerTK, CD36, CD91, Axl, Tyro3) in our models. Failure to observe fluticasone-augmented efferocytosis may therefore reflect the baseline receptor profile of healthy phagocytes, which is likely distinct from that of COPD-derived macrophages or TAMs. Future studies that directly measure receptor modulation by ICS/LABA in disease-relevant phagocytes will be essential.

### 3.3. Fluticasone-Mediated Neutrophil Survival Without Enhanced Efferocytosis

Fluticasone significantly reduced spontaneous 24-h neutrophil apoptosis by decreasing the early-apoptotic fraction, without affecting late apoptosis or necrosis. This phenotype is consistent with the established anti-apoptotic actions of glucocorticoids in neutrophils, which include inhibition of effector caspases and upregulation of anti-apoptotic Bcl-2 family proteins such as Mcl-1 and Bcl-xL [31]. We did not directly measure caspase-3/8 activity or Mcl-1/Bcl-xL expression in our neutrophil cultures, and we acknowledge this as a mechanistic limitation: the precise molecular mechanism by which fluticasone reduces early apoptosis in our system was therefore not resolved at the protein level and should be addressed in dedicated mechanistic studies.

Salmeterol alone had no effect and did not augment the survival benefit of fluticasone, in contrast to earlier reports of β2 agonist–corticosteroid synergy on neutrophil survival [32]. We interpret this difference cautiously: experimental conditions, including donor variability, the use of freshly isolated cells, and the serum-free assay format, differ substantially between studies and may account for the absence of synergy in our hands. We note that salmeterol is itself a single LABA molecule, and although LABA effects on neutrophil survival may show class behavior, formoterol-independent and salmeterol-independent actions cannot be excluded without head-to-head testing.

The observation that fluticasone prolongs neutrophil survival without augmenting efferocytosis is clinically meaningful. In the COPD airway, longer-lived neutrophils accumulate locally; if they then undergo apoptosis in a microenvironment of defective macrophage efferocytosis [2,16], they progress to secondary necrosis and release proteases, reactive oxygen species, and DAMPs that drive secondary infection and amplify the destructive inflammatory cycle of COPD. Bourbeau and colleagues demonstrated that fluticasone propionate monotherapy tended to increase airway neutrophil counts in patients with COPD, an effect attenuated by salmeterol co-treatment—consistent with the interpretation that corticosteroid-induced neutrophil persistence is modifiable by LABA co-administration in vivo [35].

### 3.4. Clinical Implications—The COPD–Lung Cancer Efferocytosis Nexus

Defective efferocytosis has substantial clinical implications at the interface of COPD and lung cancer. In COPD, impaired apoptotic-cell clearance drives secondary necrosis and release of pro-inflammatory mediators that sustain chronic airway inflammation [3]. When lung cancer arises in this milieu, TAMs are confronted both with an increased apoptotic load—generated by anticancer therapy and by spontaneous tumor-cell death—and with a pre-existing efferocytic defect. Recent work indicates that the FAS–FAS-ligand axis on apoptotic cells engages fibroblast-mediated tissue remodeling and is integrated into efferocytic outcomes [42], while mitochondrial dynamics within alveolar macrophages—including SerpinB2-dependent mitochondrial regulation—shape their inflammatory and efferocytic phenotypes [43,44]. Together, these pathways position alveolar-macrophage biology as a central determinant of the COPD–lung cancer efferocytosis nexus.

In our study, fluticasone and salmeterol did not enhance efferocytosis in healthy phagocytes in vitro. This finding suggests that, at least under acute exposure conditions, ICS/LABA therapy is unlikely to restore efferocytic clearance in COPD, where the defect is already established. Whether this dysfunction is fully irreversible in patients with COPD remains uncertain: emerging evidence indicates persistence after smoking cessation but does not exclude a contribution from reversible factors, and clinical trials of targeted efferocytic interventions have not yet been performed [16]. If conventional ICS/LABA therapy cannot correct the defect, complementary strategies targeting key efferocytic effectors—MerTK, Rac-1, MFG-E8—may be required to improve apoptotic-cell clearance and potentially modify disease progression in COPD and COPD-associated lung cancer.

ICS/LABA therapy may also influence macrophage polarization between pro-inflammatory M1 and pro-resolving M2 phenotypes. Glucocorticoids generally promote an M2-like, pro-resolving program through upregulation of CD163, CD206, and IL-10 [19,28], whereas LABA effects on polarization are less well characterized and appear context-dependent. We did not measure polarization markers in the present work; characterization of M1/M2 transitions in response to ICS/LABA in COPD- and tumor-derived macrophages is an important next step for understanding the immunological consequences of these therapies in the lung.

### 3.5. Limitations

This study has several limitations that should be considered when interpreting the results.

#### 3.5.1. Use of Tumor-Derived Cell Lines and Healthy Primary Cells

The phagocyte models comprised both healthy murine primary cells (C57BL/6 alveolar and peritoneal macrophages) and tumor-derived immortalized cell lines (J774A.1, THP-1, A549). The apoptotic target population also included a tumor-derived line (Jurkat E6.1). Crucially, none of the phagocytes were obtained from patients with COPD or from lung cancer tissue. Tumor-derived lines, although widely used and reproducible, may exhibit altered apoptosis sensing, dysregulated receptor expression, and transformed signaling that differ from primary cells. Healthy murine macrophages may not reflect the functional impairments, altered receptor profiles, and distinct transcriptional states described in COPD-derived alveolar macrophages or in TAMs within lung-cancer tissue. These constraints temper extrapolation of our findings to disease contexts.

#### 3.5.2. Serum-Free Experimental Conditions

Efferocytosis assays were performed in serum-free X-Vivo medium to avoid interference with fluorescence readouts. The absence of serum-derived opsonins (Protein S, Gas6, complement) may have prevented detection of glucocorticoid-augmented efferocytosis, which depends on these bridging molecules.

#### 3.5.3. Acute Exposure Model

Drug exposure was limited to 2–24 h, a time frame that may not capture the slower transcriptional effects of glucocorticoids on LXR, PPARδ, and efferocytic-receptor programs. In vivo, inhaled corticosteroids act over weeks to months and may produce different biological outcomes.

#### 3.5.4. No Assessment of Efferocytic-Receptor Expression

We did not measure changes in efferocytosis receptors (e.g., MerTK, Axl, Tyro3, CD36, CD91, αvβ3). Because receptor regulation is a key mechanism of glucocorticoid action, this limits mechanistic interpretation.

#### 3.5.5. No Analysis of Downstream Anti-Inflammatory or Pro-Apoptotic Mediators

We did not assess downstream cytokines (e.g., IL-10, TGF-β, PGE2) released after efferocytosis or canonical pro/anti-apoptotic proteins (caspase-3, caspase-8, Mcl-1, Bcl-xL) in the neutrophil cultures, which limits resolution of the molecular mechanisms underlying the observed neutrophil-survival phenotype.

#### 3.5.6. Limited Sample Size in Cytotoxicity Screening

Preliminary viability experiments (Figure 1) were performed with small sample sizes (n = 2–3). These results should therefore be regarded as exploratory and hypothesis-generating rather than definitive.

## 4. Materials and Methods

### 4.1. Reagents and Test Compounds

Fluticasone propionate and salmeterol xinafoate were obtained from Sigma-Aldrich (Taufkirchen, Germany) and dissolved in dimethyl sulfoxide (DMSO; Sigma-Aldrich, Taufkirchen, Germany) to prepare stock solutions. Working concentrations (10^−8^–10^−4^ M) were freshly prepared in culture medium prior to each experiment, with final DMSO concentrations not exceeding 0.1% (*v*/*v*); equivalent vehicle controls were included throughout. Functional assays were performed at 10^−8^–10^−6^ M, a range chosen to bracket the local epithelial-lining-fluid concentrations of inhaled fluticasone propionate and salmeterol xinafoate estimated from clinical pharmacokinetic studies [36,37], while 10^−5^–10^−4^ M concentrations were reserved for cytotoxicity screening (Figure 1). Dexamethasone (Sigma-Aldrich, Taufkirchen, Germany) was used at 1 µM as a reference glucocorticoid. Phorbol 12-myristate 13-acetate (PMA; Sigma-Aldrich, Taufkirchen, Germany) was used at 100 nM for THP-1 differentiation. Cell-culture media (RPMI-1640, DMEM), L-glutamine, and fetal calf/bovine serum were obtained from Sigma-Aldrich (Taufkirchen, Germany). Annexin-V–FITC and propidium iodide were obtained from Becton Dickinson (Heidelberg, Germany); Sytox Green and Hoechst H-33342 were obtained from Molecular Probes/Invitrogen (Eugene, OR, USA); Calcein AM was obtained from Thermo Fisher Scientific (Waltham, MA, USA). Polymorphprep was obtained from Axis-Shield PoC AS (Oslo, Norway).

### 4.2. Cell Lines and Primary Cells

*Tumor-derived cell lines.* All four immortalized lines used—both phagocyte models and apoptotic targets—are tumor-derived rather than healthy primary cells. The human acute T-cell leukemia line Jurkat E6.1 (American Type Culture Collection [ATCC], Manassas, VA, USA) was used as an apoptotic target; the human acute monocytic leukemia line THP-1 (Sigma-Aldrich, Taufkirchen, Germany) was used after PMA-induced differentiation as a macrophage-like model; the human alveolar adenocarcinoma (NSCLC) line A549 (ATCC, Manassas, VA, USA) served as a non-professional phagocyte model; and the murine macrophage line J774A.1 (Sigma-Aldrich, Taufkirchen, Germany), originally derived from a reticulum-cell sarcoma, served as a professional phagocyte model. The malignant origin of each line is summarized in Table 2.

#### Murine Primary Cells

Peritoneal macrophages, alveolar macrophages, and primary thymocytes were obtained from female C57BL/6 mice (8–12 weeks of age; Charles River Laboratories, Sulzfeld, Germany). Only female animals were used to limit inter-individual variability arising from sex-dependent differences in macrophage phenotype and to standardize the donor population. Peritoneal and alveolar macrophages were cultured in RPMI-1640 with 10% (*v*/*v*) FCS. Thymocytes were freshly isolated from excised thymus tissue by mechanical dissociation and filtration through a sterile cell strainer to obtain single-cell suspensions and were used immediately.

### 4.3. Human Blood Neutrophil Isolation

Human peripheral blood was purchased from the German Red Cross Blood Donor Service NSTOB (DRK-Blutspendedienst NSTOB gGmbH, Springe, Germany) as anonymized buffy-coat or whole-blood units obtained from healthy adult donors under the supplier’s standard informed-consent and ethical framework (see Informed Consent Statement). Neutrophils were isolated using Polymorphprep (Axis-Shield PoC AS, Oslo, Norway) according to the manufacturer’s instructions, as previously described [45]. Cells were counted using an automated cell counter (Nexcelom Bioscience, Lawrence, MA, USA), and the quality of isolation was assessed by cytospins stained with May–Grünwald/Giemsa.

### 4.4. Cell Viability Assessment

Cell viability and modes of cell death (apoptosis vs. necrosis) were assessed by two complementary fluorescence approaches. (1) Annexin-V–FITC/propidium iodide staining (Becton Dickinson, Heidelberg, Germany) was performed according to the manufacturer’s instructions, with detection by flow cytometry (FACSCalibur; Becton Dickinson) or fluorescence microscopy (Axiovert 200; Carl Zeiss, Oberkochen, Germany). (2) Dual Sytox Green/Hoechst H-33342 staining (Molecular Probes, Eugene, OR, USA) was performed at 1 µM and 5 µg/mL, respectively, with imaging by fluorescence microscopy: Sytox Green identifies membrane-compromised (necrotic) cells, and Hoechst H-33342 labels condensed chromatin in apoptotic cells. This combination distinguished necrotic (Sytox^+^, uncondensed nuclei), early apoptotic (intact membrane, condensed nuclei), and late apoptotic (Sytox^+^, condensed/fragmented nuclei) cells. At least 200 cells were scored per condition by an investigator blinded to treatment.

### 4.5. Induction of Apoptosis and Identification of Apoptotic Cells

Jurkat T cells were induced to undergo apoptosis by UV irradiation (100 mJ/cm^2^; CL-1000 UV Crosslinker, UVP/Analytik Jena, Upland, CA, USA) as previously described [45,46]. Apoptosis in murine thymocytes was induced by dexamethasone (1 µM, 4 h; Sigma-Aldrich, Taufkirchen, Germany). The proportions of viable, early apoptotic, late apoptotic, and necrotic cells were quantified by fluorescence microscopy after annexin-V/propidium iodide and Sytox Green/Hoechst H-33342 staining at 2, 6, and 24 h.

### 4.6. Differentiation of THP-1 Monocytes into Macrophages

THP-1 monocytes were seeded in RPMI-1640 supplemented with 10% FBS and 1% penicillin/streptomycin. Differentiation into macrophage-like cells was induced by the addition of 100 nM PMA. Cells were incubated for 72 h at 37 °C in a humidified atmosphere containing 5% CO_2_. After differentiation, PMA-containing medium was removed, cells were thoroughly washed with fresh medium to eliminate residual PMA, and were rested in PMA-free complete medium for a defined recovery period before use.

### 4.7. Efferocytosis Assay and Fluorescent Labeling

Standardized in vitro efferocytosis assays were performed as previously described [47]. Briefly, phagocytes were seeded at optimal density in IMDM (Sigma-Aldrich, Taufkirchen, Germany) and allowed to adhere overnight or for at least 4–5 h prior to the assay. Apoptotic target cells (Jurkat T cells or murine thymocytes) were fluorescently labeled with Calcein AM (1 µM, 30 min, 37 °C; Thermo Fisher Scientific, Waltham, MA, USA), then washed three times with PBS to remove unincorporated dye. Phagocyte medium was replaced with serum-free X-Vivo 15 medium (Lonza, Cologne, Germany) immediately before the assay. Labeled apoptotic targets were added to phagocyte cultures at predetermined phagocyte-to-target ratios (1:5 for macrophages, 1:10 for A549) and co-incubated with test substances (fluticasone, salmeterol, or fluticasone + salmeterol at 10^−8^ M or 10^−6^ M; dexamethasone at 1 µM) for 2 h or 24 h as specified. DMSO vehicle controls (≤0.1% *v*/*v*) were included in every experiment. After co-incubation, non-engulfed targets were removed by three PBS washes, and efferocytic uptake was quantified by fluorescence microscopy with at least 200 phagocytes scored per condition by an investigator blinded to treatment. The efferocytic index is reported as the percentage of phagocytes containing one or more Calcein AM-positive apoptotic targets.

### 4.8. Statistical Analysis

Values are expressed as mean ± standard error of the mean (SEM). Gaussian distribution of values was assessed using the Kolmogorov–Smirnov test. Comparisons between two groups were performed using Student’s *t*-test (for normally distributed data) or the Mann–Whitney U test (for non-normally distributed data). Comparisons among three or more groups were performed using one-way analysis of variance (ANOVA) followed by the Student–Newman–Keuls post hoc test. Outlier values were identified using Grubbs’ test (α = 0.05) and retained in the primary analysis; sensitivity analyses excluding outliers gave qualitatively identical results. Statistical significance was set at *p* < 0.05. All analyses were performed using GraphPad Prism version 10.0 (GraphPad Software Inc., Boston, MA, USA).

## 5. Conclusions

We evaluated the effects of fluticasone propionate, salmeterol xinafoate, and their combination on inflammatory cell survival and efferocytosis across professional and non-professional phagocyte models. Fluticasone significantly reduced spontaneous 24 h neutrophil apoptosis but did not enhance efferocytosis in any phagocyte type tested. Salmeterol showed no independent effect and did not synergize with fluticasone in this acute, serum-free model. We therefore reject the a priori hypothesis that clinically relevant concentrations of fluticasone and salmeterol—alone or in combination—augment efferocytic clearance under acute in vitro conditions, while confirming the established neutrophil pro-survival action of fluticasone. Given the central role of defective efferocytosis in COPD and its emerging contribution to lung-cancer pathogenesis, our findings highlight a potential limitation of current inhaled therapies in restoring this homeostatic pathway. Further studies in disease-relevant phagocyte populations—including alveolar macrophages from patients with COPD and TAMs from lung-cancer tissue—and in vivo models will be required to determine whether pharmacological targeting of the efferocytic machinery itself can deliver clinical benefit in COPD and COPD-associated lung cancer.

## Figures and Tables

**Table 1:** Quantitative summary of efferocytic activity across phagocyte models.

Phagocyte	Apoptotic Target	Co-Incubation Time	Baseline Efferocytosis (Vehicle)	Fluticasone (10^−8^–10^−6^ M)	Salmeterol (10^−8^–10^−6^ M)	Flu + Sal	ANOVA *p*	n	Figure
*Murine peritoneal macrophages*	Apoptotic thymocytes	2 h	~75%	No change	No change	No change	0.92	3	6
Murine alveolar macrophages	Apoptotic thymocytes	2 h	~40%	Non-significant ↑	Non-significant ↑	Non-significant ↑	0.26	4	5
J774A.1 (murine line)	Apoptotic Jurkat	24 h	~75%	No change	No change	No change	n.s.	4	7
PMA-differentiated THP-1 (human)	Apoptotic Jurkat	2 h	~30%	No change	No change	No change	0.91	4	8A
PMA-differentiated THP-1 (human)	Apoptotic Jurkat	24 h	~60%	No change	No change	No change	0.96	3	8B
A549 (human, non-professional)	Apoptotic Jurkat	24 h	<20%	No change	No change	No change (Dex also no effect)	0.68–0.88	4	9

n.s., not significant. Values are mean efferocytic indices from independent experiments; full distributions are shown in the corresponding figures. The arrow symbol (↑) denotes a non-significant numerical trend toward increased efferocytosis relative to the vehicle control (*p* ≥ 0.05, one-way ANOVA).

**Table 2:** Tumor origin of immortalized cell lines used in this study.

Cell Line	Species	Tissue/Disease of Origin	Role in This Study
Jurkat E6.1	Human	Acute T-cell leukemia (peripheral blood)	Apoptotic target cell
THP-1	Human	Acute monocytic leukemia	Macrophage-like phagocyte (after PMA)
A549	Human	Alveolar adenocarcinoma (NSCLC)	Non-professional phagocyte
J774A.1	Mouse	Macrophage line from reticulum cell sarcoma	Professional phagocyte

Jurkat E6.1 cells were cultured in RPMI-1640 supplemented with 10% (*v*/*v*) fetal calf serum (FCS). THP-1 cells were cultured in RPMI-1640 with 2 mM L-glutamine and 10% (*v*/*v*) FCS. A549 cells were cultured in RPMI-1640 with 10% (*v*/*v*) FCS. J774A.1 cells were cultured in high-glucose DMEM with 2 mM L-glutamine and 10% fetal bovine serum (FBS).

## Data Availability

The original contributions presented in this study are included in the article. Further inquiries can be directed to the corresponding author.
